# A UK study: vocational experiences of young adults with juvenile idiopathic arthritis

**DOI:** 10.1186/s12969-019-0357-y

**Published:** 2019-08-06

**Authors:** Laura E. Lunt, Ailsa Bosworth, Matthew Bezzant, Karen Walker-Bone, Kimme L. Hyrich, Wendy Thomson, Janet E. McDonagh, Suzanne M. M. Verstappen

**Affiliations:** 1 Centre for Epidemiology Versus Arthritis, The University of Manchester, Manchester, UK; 2 NIHR Manchester Biomedical Research Centre, Manchester Academic Health Science Centre, Manchester University Hospitals NHS Foundation Trust,, Manchester, UK; 3 National Rheumatoid Arthritis Society, (NRAS), Maidenhead, UK; 4 Arthritis Research UK/MRC Centre for Musculoskeletal Health and Work, University of Southampton, Southampton, UK; 5 Centre for Genetics and Genomics Versus Arthritis, The University of Manchester, Manchester, UK

**Keywords:** Transitional care, Vocational development, Young people, Rheumatic and musculoskeletal diseases

## Main text

We commend Dr. Walter and colleagues [1] for their evaluation of a clinical transition pathway for young people with rheumatic and musculoskeletal diseases. Their work highlights a successful health transition model and as indicated in their findings, addresses some of the EULAR/PReS transitional care recommendations [2]. However we were interested to read the significant proportions of young people reporting a negative impact of their condition on education/vocational aspects of their lives [1]. By definition, transitional care should address vocational as well as medical, social and psychological issues as young people move into adult services.

We would like to draw your attention to our recent UK survey of 16+ year olds on this topic which included 19 young adults, (*n* = 17 female *n* = 17 white British) diagnosed with juvenile idiopathic arthritis [3]. Young people participating in a national survey, when in education, reported a lack of careers guidance. 8/14 respondents felt their school careers adviser did not take their condition into account when providing careers information/advice. Respondents reported the lack of support in schools for students with additional needs in planning their work experience; for example, 10/14 respondents reported a lack of information on possible limitations they may encounter on a work experience placement/traineeship (Table 1).

**Table 1:** Work experience (N)

	Strongly agree	Agree	Neither agree nor disagree	Disagree	Strongly disagree	Don’t know
My school offered a placement which matched my career ambitions	2	6	2	3	1	0
My school looked at my strengths and matched these skills with an employer willing to take on these skills	1	5	4	2	2	0
My school encouraged me to try any work despite possible physical limitations due to my arthritis	1	4	5	2	2	0
My school matched my placement/traineeship based on my studies subjects	3	5	3	1	2	0
My school identified placements that stretched/challenged my view of work	2	2	6	3	1	0
My work experience placement/traineeship was easily accessible or near home	6	4	2	2	0	0
My school provided advice about possible limitations about some placements or traineeships due to my arthritis	1	0	2	7	3	1

Young people do take their condition into consideration when planning a career. 8/14 respondents changed their career path because of their condition reflecting the results reported by Walter et al. [1]. Reasons for this included the need to stay healthy, physical demands or to manage symptoms. It is unclear in our study if these changes were seen as positive or negative. However, as highlighted by Walter et al., some young people with rheumatic diseases perceive their condition to have a negative effect on their chosen vocation/career [1].

The EULAR/PReS standards highlight the need to implement a holistic approach to care, which includes vocational and career planning [2]. However, a study evaluating European paediatric rheumatology transitional care practice, found that approximately 36% used a transitional care checklist and only 50% of these addressed vocational readiness [4].

As indicated by Walter et al. and others [1, 5], interventions are needed to better support young people with rheumatic conditions to improve transition outcomes. Further research in the UK is needed to explore how such transitional care can improve vocational outcomes. Finding the right first job is likely to influence young people’s future employment prospects and career opportunities.

## Data Availability

The datasets generated and analysed during the current study are not publicly available, as consent to share publicly was not obtained by participants.

## Abbreviations

EULAR: European League Against Rheumatism
PReS: Pediatric Rheumatology European Society
